# How Did Work-Related Depression, Anxiety, and Stress Hamper Healthcare Employee Performance during COVID-19? The Mediating Role of Job Burnout and Mental Health

**DOI:** 10.3390/ijerph191610359

**Published:** 2022-08-19

**Authors:** Jianmin Sun, Muddassar Sarfraz, Larisa Ivascu, Kashif Iqbal, Athar Mansoor

**Affiliations:** 1 School of Management, Nanjing University of Posts and Telecommunications, Nanjing 210006, China; 2 School of Management, Zhejiang Shuren University, Hangzhou 310015, China; 3 Faculty of Management in Production and Transportation, Politehnica University of Timisoara, 300191 Timisoara, Romania; 4 School of Business, Shanghai Dianji University, Nanhui Xincheng Town, Shanghai 201306, China; 5 Division of Public Policy, Hong Kong University of Science and Technology, Hong Kong

**Keywords:** stress, anxiety, depression, psychological well-being, COVID-19, mental health, job burnout

## Abstract

The study objective was to examine the psychological impact of the COVID-19 pandemic on the performance of healthcare employees. The study was informed by a theoretical framework that incorporates different psychological issues (i.e., stress, depression, and anxiety) that influence healthcare workers’ performance through the mediating roles of job burnout and mental health. The study data was gathered through structured questionnaires from 669 participants working in the healthcare sector in Pakistan. A structured equation modeling (SEM) technique was used for data analysis and hypothesis development. It was found that stress, depression, and anxiety positively affected healthcare employees’ job performance during COVID-19. Psychological factors had a positive and significant impact on job burnout and mental health. Job burnout and mental health mediated the relationship between stress, anxiety, depression, and employee performance. The ongoing repercussions of COVID-19 include their impact on employee performance in the healthcare sector. Healthcare worker performance is critical to fostering industrial economic growth. Elevated levels of stress, depression, and anxiety have profoundly exacerbated employee mental health issues. COVID-19 has created challenging working conditions in organizations requiring that they address the growing psychological issues which impact negatively on worker performance.

## 1. Introduction

In recent years, the emergence of the COVID-19 pandemic has demonstrated how a new virus can significantly alter human life. The profound changes caused by COVID-19 have presented major social challenges across the world with significant effects across a variety of domains [1]. The prolonged nature of the crisis, and its widespread impact, has led to its declaration as a global health emergency. The sudden onset of the COVID-19 pandemic caused psychological distress across industries throughout the world [2]. Notably, the very significant mental health impacts arising have exacted an extreme toll on the healthcare industry, with a particularly severe impact on healthcare performance.

For these reasons, COVID-19 has become a global threat to healthcare performance [3]. The prevalent psychological problems occurring in the healthcare sector can be partly attributed to the disease’s ability to cause previously healthy employees to become vulnerable caretakers. COVID-19 has also exacerbated existing mental health problems among healthcare employees due to the psychological trauma and distress they face in their work. Research has shown that front-line workers in the healthcare sector (e.g., nurses, doctors, medical staff, and health professionals) are presently facing unprecedented psychological challenges [4].

In dealing with these exacerbated health vulnerabilities, Pakistan’s economy has experienced a setback with front-line workers exposed to significant risks to their health. A recent study from Pakistan found that the COVID-19 crisis meant that healthcare workers had experienced increasing psychological pressure, leaving them ill-equipped to tackle the increasing challenges they faced [5]. The virus has caused healthcare workers to experience significant psychological repercussions, which have detrimentally affected their job performance. The crisis has created an enhanced sense of helplessness in health professionals, that can further deteriorate employee performance [6]. The literature demonstrates, however, that, despite growing evidence of mental health problems, the healthcare workforce is not seeking the mental healthcare necessary to ensure their well-being and performance [7,8]. Therefore, to limit the impact of COVID-19, it has become imperative for global healthcare institutions to recognize their responsibility toward their workers’ well-being, specifically regarding the increase in the COVID-19-related psychological burden. Previous studies have emphasized the need for multidisciplinary research in the healthcare sector concerning the growing psychological issues that influence employee mental health and job performance.

This global threat of COVID-19 has impacted the psychological health of individuals, with outbreaks representing stressful events for front-line workers. The COVID-19 crisis provoked nations globally to take action to combat the devastating spread of the pandemic. The authors of one study observed that the significant repercussions of COVID-19 have damaged employees’ psychological well-being, thereby negatively impacting their normal intellectual functioning [9]. Overall, the pandemic has overwhelmed the healthcare sector by reducing employees’ work performance [10,11].

The COVID-19 pandemic has gravely impacted the psychological health of individuals, causing them to experience significant mental health issues. The ongoing crisis has negatively impacted employees, making them psychologically ill-prepared to perform their normal tasks. Further, the situation has exacerbated existing psychological problems (e.g., stress and anxiety) [12], substantially impeding individuals’ job performance. Aguiar-Quintana et al. [13] found that high levels of COVID-19 exposure led to stress, anxiety, and depression symptoms, thus influencing workers’ performance. Overall, the pandemic has imposed significant strain on an already traumatized workforce, with increased anxiety and tension further exacerbating its impact on healthcare performance [14].

Employee mental health during the pandemic has also increased the burnout rate in the healthcare industry. Accelerating absenteeism and turnover intentions among healthcare employees caused by COVID-19, with high burnout rates reported, have had very damaging consequences,. Unsurprisingly, job burnout detrimentally impacts individuals’ mental health [15], thereby hampering their performance. Factors contributing to increasing burnout increase workplace mental pressure, affecting healthcare workers’ ability to perform their work tasks [16].

Therefore, to address the negative consequence of the pandemic, this study sought to investigate the effect of COVID-19 on healthcare workers in Pakistan. This paper presents evidence of the adverse effects of COVID-19 in the light of previous literature. The paper highlights the harsh realities of fighting the virus, and the need to safeguard healthcare employees against the psychological impacts of the pandemic and to prepare them physically and psychologically. Previous studies have investigated the clinical characteristics of the pandemic, its negative features, and the health measures implemented. In this regard, this study represents a valuable addition, providing information to help address the uncertainties of an unprecedented pandemic situation. Adding to previous studies that have considered the psychological and mental health impacts of COVID-19, this study provides a unique perspective by considering the role of both mental health and job burnout. The study provides essential information by highlighting these vital factors to increase awareness regarding the impact of COVID-19 on health workers’ mental health and performance.

The study calls for urgent action to mitigate the devastating effects of COVID-19 on employee performance and psychological health. The study findings provide vital tools to combat the increasing impact of stress, anxiety, and depression influencing healthcare performance. It provides a concrete basis for adapting and executing appropriate mental health policies to address the psychological vulnerabilities generated by COVID-19. It encourages government bodies, health professionals, and policymakers to protect the psychological well-being of healthcare workers in different parts of the world, specifically in Pakistan.

## 2. Theoretical Background

### 2.1. Stress and Employee Performance

In recent years, globalization and technological advancement have enhanced individuals’ working standards. However, the sudden outbreak of the COVID-19 pandemic has altered the working environment and job demands. In recent years, this profound crisis has made working conditions difficult, significantly raising organizations’ concerns regarding employee management. Yunita and Saputra [17] found that stress is the foremost factor that has influenced employee functioning during the pandemic. Stress has adversely affected individuals’ morale, performance, and motivation. In particular, the negative changes caused by COVID-19 have altered healthcare workers’ lives by significantly impeding their work performance. Prasada et al. [18] demonstrate that the growing pandemic stress has created a sense of chaos, leading organizations to report poor worker performance.

Various studies have reported that stress is the prime determinant influencing employee performance [19]. The pandemic has caused healthcare employees to experience workplace stress to an unprecedented extent. The increasing COVID-19 stress has exerted intense pressure on healthcare workers by creating additional job demands. The literature suggests that employees who experience stress tend not to meet job expectations. In the healthcare industry, the crisis situation has increased stress symptoms in frontline workers, with detrimental impacts on employee output. Tu et al. [20] observed that COVID-19-induced stress has influenced individuals’ ability to perform well, leading to substantial decreases in employees’ quality of work. 

The occupational stress experienced during the pandemic outbreak has significantly influenced the economic functioning of nations, necessitating a focus on workers’ job performance. Evidence provided in the literature indicates that stress occurring as a result of the COVID-19 pandemic is prevalent as an issue affecting healthcare employee performance [21]. An employee experiencing a high degree of stress has lower motivation to perform the task. The COVID-19 crisis has caused individuals’ to focus less well on work-related tasks, substantially reducing their overall work performance. The pandemic has affected the flow of work, potentially increasing individuals’ workload. The perceived work burden has elevated stress in individuals, resulting in a decrease in individuals’ work performance [22]. 

In sum, the uncontrollable nature of COVID-19 has negatively affected healthcare workers, provoking anxiety and stress. COVID-19 has caused increasing uncertainty, with healthcare staff reporting psychological symptoms, emotional exhaustion, and workload stress. As the pandemic worsened, these symptoms accelerated, causing healthcare workers to face increased traumatic stress [23]. The issue of social stigmatization and shortages of healthcare equipment have made it difficult for employees to deal with the impacts of COVID-19 [24]. As this industry has faced a particularly marked increase in stress and depression during the pandemic, it is important to focus on the health of Pakistan’s healthcare workers to ensure enhanced work output.

### 2.2. Depression and Employee Performance

In recent years, the growing strain of the pandemic has encircled the globe, negatively impacting countries across the world. The COVID-19 outbreak has impacted healthcare services internationally, including in the Pakistan healthcare sector. The aggregate effect of the pandemic has meant healthcare employees have experienced an increasing intensity of demands due to the crisis, giving rise to the need for immediate investment in healthcare recovery. Healthcare workers are at risk of being affected psychologically by the pandemic situation. Depression has emerged as a harmful outcome that hinders employee functioning [25]. In healthcare, depression has become a major obstacle to employee performance. Healthcare employees are vulnerable to depression during the pandemic because of their exposure to various psychological stressors. Examination of mental responses related to COVID-19 has confirmed that depression drastically impedes healthcare performance. One study demonstrated how intense workloads occurring during the pandemic have increased depression among individuals, in turn negatively affecting work quality [26,27].

The pandemic has resulted in significant changes in the work environment of healthcare workers. The pandemic has led to worsened working conditions, causing employees to be more vulnerable to depression and distress. The elevated psychological pressure has raised management concerns, leading to demands for a reduction in employee workloads. Studies on the causes of poor healthcare performance suggest that the alarming COVID-19 situation has meant that employees are at risk of depression, impeding their performance [28,29]. Depression considerably affects the work status of healthcare workers, overwhelming them and negatively impacting their work performance. Research findings indicate that depression results in poorer work outcomes, negatively affecting healthcare performance [30]. Research into the impacts of the pandemic suggests that measures must be taken so that healthcare employees can deal with the increasing depression that can influence their productivity. 

### 2.3. Anxiety and Employee Performance

COVID-19 has altered the typical working patterns of healthcare workers and significantly affected the psychological well-being of medical staff. Combating this new virus, initially without proven prevention measures or treatments, imposed a significant burden on the medical workforce. This situation has required that organizations globally take care of their workers and protect them against the psychological effects of their vulnerability to COVID-19 exposure. Despite awareness of this need, healthcare workers have been significantly affected across the globe. One empirical study found that around 53.8% of healthcare employees have been diagnosed with psychological issues [21]. Additionally, 21.3% of healthcare workers have experienced anxiety [31]. Results of studies from the Asian region indicate that, currently, healthcare employees are experiencing a high level of stress, anxiety, and depression symptoms [32].

Depression and anxiety significantly influence individuals’ professional lives, and coping with anxiety has become a widespread challenge in today’s world. In the era of COVID-19, increasing work anxiety has led to significant impacts on the healthcare industry. The pandemic has increased anxiety in individuals, necessitating the study of its effect on individuals’ job performance. Work anxiety experienced as a result of the COVID-19 situation can potentially influence healthcare performance. For example, Fu et al. [33] found that prevalent COVID-19 anxiety heightened job-related concerns, substantially diminishing employees’ healthcare performance. 

Several factors affect employee performance, but among them, anxiety is a critical factor that demands the attention of researchers. Due to the uncertainty brought about by the pandemic, employee anxiety has had a devastating impact as a result of changed working environments, contributing to reduced work performance. Clercq et al. [34] suggested that anxiety associated with the pandemic has elevated job-related worries in healthcare workers, causing them to exhibit poor performance. The negative influence of COVID-19 has caused employees to experience excessive tension and anxiety, particularly in the healthcare sector. Anxiety has been demonstrated to have a toxic effect on employee performance, with Kumar et al. [35] stating that the high prevalence of anxiety and depression during the pandemic has undermined the performance of the healthcare workforce. Consistent with this discussion, Nadeem et al. [36] observed that the healthcare workforce had encountered severe anxiety, making it difficult for individuals to cope with work complexities brought about by COVID-19. 

### 2.4. The Mediating Role of Job Burnout

Burnout is a global phenomenon and has been exacerbated as the world’s healthcare industry has faced the growing consequences of the pandemic. In 2020 and 2021, the progressive impact of COVID-19 has generated burnout. Inevitably, the pandemic has negatively influenced the lives of healthcare workers. Since the beginning of 2020, the Pakistan health workforce has had to cope with a wide range of crises, leading to job burnout. A recent study from Pakistan indicated that around 46.6% of healthcare workers have left their job due to the pandemic [37]. Healthcare burnout is an accelerating phenomenon that has raised awareness of the need to find solutions to combat COVID-19 work stress and job burnout [38]. Bradley and Chahar [39] found that burnout has drastically increased during the pandemic years, emphasizing the need to provide immediate support.

The emergence of a fourth wave of COVID-19 saw anxiety and stress rapidly increase in Pakistan. The wide circulation of the virus has placed a psychological burden on healthcare workers, causing them to experience an increased level of burnout [40]. The uncertainty surrounding the outbreak has increased concerns about healthcare workers and their rate of burnout [41]. Despite the ongoing nature of the crisis, Pakistan still lacks the medical equipment needed to function effectively in this difficult situation. In this regard, medical support for healthcare workers is urgently required to maintain superior quality services [42], and to ensure employees’ intention to stay in the sector. During the pandemic, the accelerating incidence of burnout has exacerbated the negative effect of healthcare workers’ psychological conditions. In the current period of the pandemic, the public health emergency has caused the healthcare workforce to experience numerous physical and psychological issues, leading to excessive employee burnout. Employee burnout has been the most prevalent negative health outcome observed in the healthcare industry in the past few years. As a result, today, the increasing impact of burnout has caused health professionals to be less focused on their work performance [43,44].

The outbreak of COVID-19 has significantly affected the working lives of healthcare workers. In recent years, these issues have strongly increased the turnover rate. The high prevalence of stress, anxiety, and depression have clearly caused burnout in healthcare employees [45]. The psychological adversity experienced has influenced healthcare employees‘ job performance, leading some to give up their profession [46]. Saleem et al. [47] found that, faced with the excessive burden of the pandemic, pandemic stress and depression have caused healthcare employees to leave healthcare organizations, substantially impeding the ability of these organizations to perform their role.

Healthcare employees have experienced reduced work productivity due to the pandemic, thus leading to a higher turnover rate. One study found that, in the healthcare sector, reduced work performance had elevated the feeling of negativity (e.g., stress, anxiety) in individuals, increasing the overall turnover rate [48]. Further, in a previous systematic review, it was found that, in the COVID-19 era, health professionals frequently experience burnout with consequent reduced job performance [49]. Long-term stress and anxiety have become critical factors leading to the increase in the burnout rate among healthcare workers [50].

### 2.5. The Mediating Role of Mental Health

In the current pandemic scenario, employees’ organizational performance has been impacted by COVID-19′s effect on individuals’ mental health [51]. Organizations strive to sustain themselves in this competitive world by empowering employees to perform well. To achieve this goal, organizations emphasize maintaining positive mental health for superior work performance. Satici et al. [52] state that the negative consequences of the COVID-19 pandemic have created a high level of anxiety, stress, and depression in individuals, thus hindering their healthcare performance.

Arguably, various factors affect the psychological well-being of workers. Among them, stress, depression, and anxiety hold a prominent position in impeding employee work performance. As the pandemic unfolds, numerous mental health problems have been highlighted, demanding researchers’ attention [47]. An employee’s mental health considerably influences their work performance. During COVID-19, workplace changes have made healthcare employees exhibit poorer work performance. Stress, depression, and anxiety are the most prominent psychological issues that have emerged as the dominant threats to employee well-being and performance [53]. Mental health issues, such as stress, depression, and anxiety affect the performance of front-line workers, as shown by Lei et al. [54].

COVID-19-related psychological problems (e.g., distress and depression) threaten healthcare employees’ mental health, and result in poor work performance [17]. In particular, Lai et al. [55] showed that, in the nursing profession, the excessive pandemic workload posed a threat to work efficiency and productivity. Therefore, the literature has highlighted the need to analyze the psychological factors that influence mental health and performance in the workplace. 

**Figure 1:**
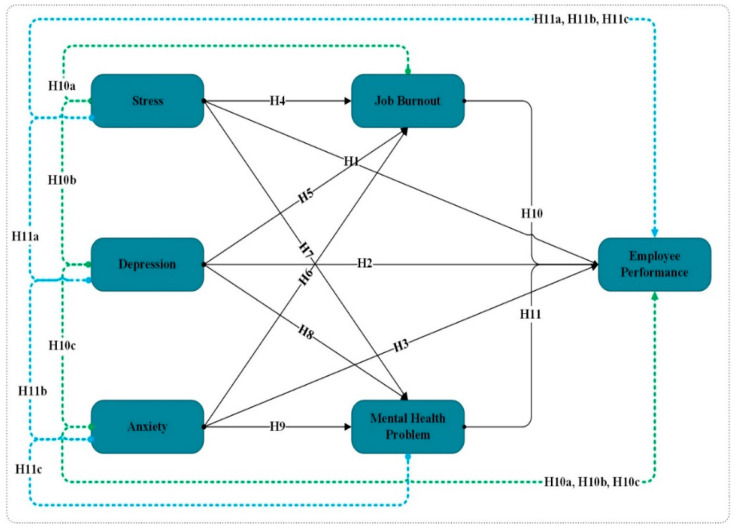
Conceptual Framework.

Figure 1 represents the direct and indirect relationships among the study variables (stress, depression, anxiety, job burnout, mental health problems and employee performance).

## 3. Methods

### 3.1. Study Procedure

A quantitative research design using a self-reported questionnaire was used to collect data from healthcare employees of 30 hospitals in Pakistan. A purposive sampling technique was used to gather data from healthcare employees from January to March 2022. The healthcare workers sampled were treating COVID-19 patients. In this study, we considered government COVID-19 treatment facilities located in three major cities of Pakistan (Lahore, Islamabad, and Karachi). To design the online questionnaire, a public platform recommended by Google INC: Google Docs was used, and the survey link was sent to healthcare employees. It was confirmed through a confidential statement that the personal information of participants would be protected and that the responses provided would only be used for research purposes. In accordance with the Declaration of Helsinki, an information letter was provided and an informed consent form obtained from the study participants. 

### 3.2. Common Method Bias

This study addressed common method bias using Harman’s single-factor methodology. The variance extracted using one factor was 20.900% which was less than 50%. This indicated that there was no common method bias [56].

### 3.3. Measures

Depression, anxiety, and stress were assessed using seven-item scales adopted from Vignola and Tucci [57]. Sample items included, “I felt like I was being a little too emotional/sensitive”, “I was intolerant of the things that kept me from continuing to do what I had been doing”, and “I knew my heartbeat had changed even though I hadn’t done anything physically rigorous (for example: increased heart rate, irregular heartbeat)”. In this study, the depression, anxiety, and stress scales had Cronbach’s alphas of 0.896, 0.902, and 0.897, respectively. 

Job burnout was assessed using a three-item scale adopted from Ninaus [58]. Sample items included, “I feel used up at the end of a workday”, and “I feel burned out from my work”. The job burnout scale had a Cronbach’s alpha value of 0.773 in this study. 

Mental health problems were assessed on a 15-item scale adopted from Sharma and Devkota [59]. Sample items included in the questionnaire were, “Have you been less confident than before?”, “Do you use alcohol or other substances that are causing problems in your daily life? and “Have you been anxious, restless, or having multiple worries and doubts in mind more than usual?”. The mental health problems scale had a Cronbach’s alpha value of 0.945. 

Employee performance was assessed using a 16-item scale adopted from Ferozi and Chang [60]. The Cronbach’s alpha value for the employee performance scale was 0.945; the sample items included, “I give advanced notice when unable to come to work”, “I take action to protect the organization from potential problems”, and “I perform tasks that are expected of me”.

### 3.4. Statistical Analysis

The data was analyzed using the Statistical Package for the Social Sciences (SPSS) and the Analysis of Moment Structures (AMOS) software. In this study, structural equation modeling (SEM) was used to analyze multivariate causal associations. Confirmatory factor analysis (CFA) was carried out to assess the internal validity of the model.

## 4. Results

Table 1 shows the demographic details of the study participants. Of 669 collected questionnaires, 311 useful responses were received from the male participants (46.5%) and 358 from the female participants (53.5%). Therefore, the study sample comprised an approximately equal proportion of male and female respondents. In terms of age, 92 (13.8%) respondents were 19–30 years old, 182 (27.2%) were 31–40, 158 (23.6%) were 41–50, 141 (21.1%) were 51–60, and 96 (14.3%) of the respondents were more than 60 years old. Regarding educational level, 130 (19.4%) had an intermediate degree, 216 (32.3%) had a bachelor’s degree, 240 (35.9%) had a master’s degree, and 83 (12.4%) had MPhil/other qualifications. With respect to marital status, 118 (17.6%) of the respondents were single, while 551 (82.4%) were married. 

### Assessment of Model Fit and Measurement Model

As shown in Table 2, the results of the model fit indicated that the overall measurement model provided an adequate fit of the data with all 55 items, with Chi-square = 1454.113 and df = 1415. The value of GFI was 0.929; greater than the recommended value of 0.9 as recommended by Hoyle (1995). Based on the CFI, TLI and IFI indices having values greater than the cut-off value of 0.9 (0.998; 0.998; and 0.932, respectively), the model was inferred to represent a good fit of the data [60,61]. The root mean square error of approximation (RMSEA) was 0.006, which was below the threshold of 0.08 recommended by Steiger [62]. Further, the standardized root mean squared residual (SRMR) was 0.025, which was below the threshold of 0.08 recommended by Hu and Bentler [63]. Additionally, the relative CMIN/df was 1.028, which, at less than five, indicated a good fit of the model [61].

As presented in Table 2, the results of an assessment of the standardized factor loadings of the model’s items indicated that the initial standardized factor loadings of all 55 items were above 0.6, as recommended by Hair [69], ranging from 0.692 (for EP_14) to 0.771 (STR_6).

Each of the constructs was evaluated for reliability after the uni-dimensionality of the constructs was determined. The average extracted variance (AVE), construct reliability (CR), and Cronbach’s alpha were used to evaluate reliability. The AVE results are presented in Table 2; all the values were higher than 0.5, as recommended by Nunnally and Bernstein [70], ranging between 0.52 (for employee performance) to 0.567 (for anxiety). 

The CR value, which indicates the degree to which the construct indicators reflect the latent construct, exceeded the recommended value of 0.7 for all constructs, as recommended by Bagozzi and Yi [71], ranging between 0.773 (for job burnout) and 0.945 (for mental health problems). The Cronbach’s alpha value, which describes the degree to which a measure is error-free, ranged between 0.773 (for job burnout) and 0.945 (for mental health problems), which were above the threshold of 0.7 recommended by Nunnally and Bernstein [70].

The correlation between the mental health problems scale scores and the employee performance scale scores was -0.601. The correlation between the job burnout and the mental health problems scale scores was 0.681. The data presented in Table 3 highlight that both were less than the threshold of 0.85 [72]. The results also revealed, as shown in Table 3, that the value of the off-diagonal items was lower than the value of the square root of AVE on the diagonal. This supported the view that each latent construct measurement was discriminating relative to the others, according to the Fornell–Larcker interpretation [73,74].

The descriptive statistics of the constructs are also provided in Table 3. These statistics include the mean and standard deviation. Evaluating the data presented in this table, it is evident that the highest mean value was 3.65, recorded for employee performance, and the lowest mean value was 3.59, recorded for depression. The highest standard deviation value was 0.886, for measurement of burnout, and the lowest standard deviation value was 0.785, for measurement of mental health problems.

In predicting employee performance, stress had a significance value below 0.001, as highlighted in Table 4. For this relationship, a *t*-value of −5.541 and a *p*-value were obtained.

**H1.**  *Stress has a negative and significant impact on employee performance.*

As shown in Table 4, H1 was supported because the regression weight for stress in the prediction of employee performance was significantly different from zero at the 0.001 level (two-tailed). A negative relationship was highlighted because the standardized path coefficient was −0.205. So, employee performance decreased by 0.205 standard deviations when stress increased by 1 standard deviation.

**H2.**  *Depression has a negative and significant impact on employee performance.*

There is a less than 1% chance of obtaining a t-value that is as large as the observed −3 in absolute value. With a standardized path coefficient of −0.123, the effect of depression on employee performance was, thus, negative and significant at the 0.01 level. Following these arguments, H2 was supported.

**H3.**  *Anxiety has a negative and significant impact on employee performance.*

There is a less than 5% chance of obtaining a t-value as large as the observed −2.897 in absolute value. With a standardized path coefficient of −0.113, the effect of anxiety on employee performance was, thus, negative and significant at the 0.05 level. As a result, H3 was confirmed.

**H4.**  *Stress has a positive and significant impact on job burnout.*

There is a less than 0.1% chance of obtaining a t-value as large as the observed 9.938 in absolute value. In other words, with a standardized path coefficient of 0.318, the impact of stress on job burnout was positive and significant at the 0.001 level. As a result, H4 was confirmed.

**H5.**  *Depression has a positive and significant impact on job burnout.*

There is a less than 0.1% chance of obtaining a t-value as high as the observed 11.871 in absolute value. In other words, with a standardized path coefficient of 0.368, the relationship between depression and job burnout was positive and significant at the 0.001 level. As a result, H5 was confirmed.

**H6.**  *Anxiety has a positive and significant impact on job burnout.*

There is a less than 0.1% chance of obtaining a t-value as high as the observed 8.968 in absolute value. In other words, with a standardized path coefficient of 0.278, the relationship between anxiety and job burnout was positive and significant at the 0.001 level. As a result, H6 was confirmed.

**H7.**  *Stress has a positive and significant impact on mental health problems.*

There is a less than 0.1% chance of obtaining a t-value as large as the observed 6.950 in absolute value. In other words, with a standardized path coefficient of 0.278, the impact of stress on mental health problems was positive and significant at the 0.001 level. As a result, H7 was confirmed.

**H8.**  *Depression has a positive and significant impact on mental health problems.*

There is a less than 0.1% chance of obtaining a t-value as high as the observed 7.541 in absolute value. In other words, with a normalized path coefficient of 0.279, the relationship between depression and mental health problems was positive and significant at the 0.001 level. As a result, H8 was confirmed.

**H9.**  *Anxiety has a positive and significant impact on mental health problems.*

Anxiety had a substantial positive impact on mental health problems at the 0.001 level with a standardized path coefficient of 0.341. Therefore, H9 was supported.

**H10.**  *Job burnout has a negative and significant impact on employee performance.*

Employee performance was adversely affected by job burnout at the 0.001 level with a standardized path coefficient of −0.195. Therefore, H10 was supported

**H11.**  *Mental health problems have a negative and significant impact on employee performance.*

The results indicated that the effect of mental health problems on employee performance was negative and significant at the 0.001 level with a standardized path coefficient of −0.183. Therefore, H11 is supported.

The results indicated that the most important determinants of job burnout, mental health problems, and employee performance were stress (β = −0.205), depression (β = 0.368), and anxiety (β = 0.341).

Table 5 shows that the *p*-values obtained were less than the standard level of 0.05. All hypothesized mediation effect paths were determined to be statistically significant, as shown in Table 5. Hence, hypotheses H10a, H10b, H10c, H11a, H11b, and H11c, were all supported. The next subsections explain the path analysis findings in relation to the mediation effect hypotheses.

**H10a.**  *Job burnout mediates the relationship between stress and employee performance.*

The bootstrapping results revealed that the indirect effect of stress on employee performance through job burnout was negative and significant at the 0.001 level (β = −0.062, *p* < 0.001), the 95% confidence interval (CI) using a 5000 bootstrap sample did not include 0, and the CIs were −0.058 and −0.019. The results indicate that job burnout partially mediated the association between stress and employee performance. Thus, H10a was supported.

**H10b.**  *Job burnout mediates the relationship between depression and employee performance.*

The bootstrapping results revealed that the indirect effect of depression on employee performance through job burnout was negative and significant at the 0.01 level (β = −0.072, *p* < 0.01, CI = 95%, CI-LL = −0.077, CI-UL = −0.031). The results indicate that job burnout partially mediated the association between depression and employee performance. Thus, H10b was supported.

**H10c.**  *Job burnout mediates the relationship between anxiety and employee performance.*

The bootstrapping results revealed that the indirect effect of anxiety on employee performance through job burnout was negative and statistically significant at the 0.001 level (β = −0.054, *p* < 0.001, CI = 95%, CI-LL = −0.059, CI-UL = −0.020). The results indicate that job burnout partially mediated the association between depression and employee performance. Thus, H10c was supported.

**H11a.**  *Mental health problems mediate the relationship between stress and employee performance.*

The bootstrapping results showed the indirect effect of stress on employee performance through mental health problems was negative and significant at the 0.01 level (β = −0.051, *p* < 0.01, CI = 95%, CI-LL = −0.068, CI-UL = −0.027). Mental health problems partially mediated the association between stress and employee performance. Hence, H11a was supported.

**H11b.**  *Mental health problems mediate the relationship between depression and employee performance.*

Table 5 shows the bootstrapping results, which indicate that the indirect effect of depression on employee performance through mental health problems was negative and significant at the 0.01 level; β = −0.051, *p* < 0.01, CI = 95%, CI-LL = −0.058, and CI-UL = −0.022. Mental health problems partially mediated the relationship between depression and employee performance. Thus, H11b was supported.

**H11c.**  *Mental health problems mediate the relationship between anxiety and employee performance.*

**Figure 2:**
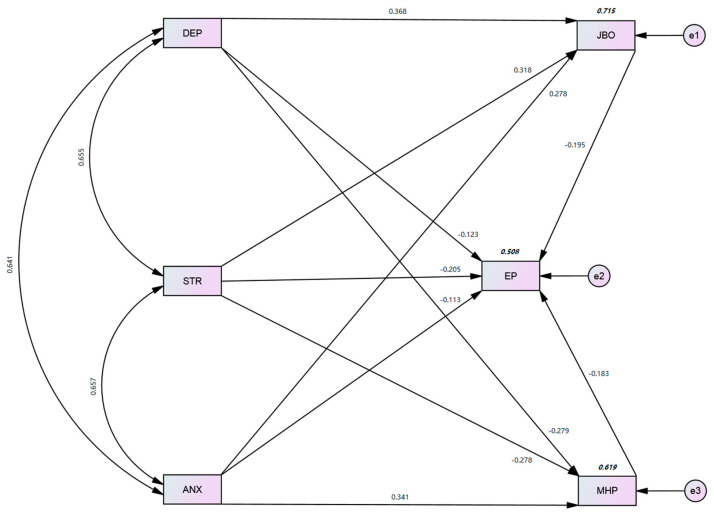
Structural Model.

Table 5 and Figure 2 shows the bootstrapping results, which indicate that the indirect effect of anxiety on employee performance through mental health problems was negative and statistically significant at the 0.01 level; β = −0.054, *p* < 0.01, CI = 95%, CI-LL = −0.071, and CI-UL = −0.021. These results, along with the significant effect of anxiety on employee performance (from Table 4), suggest that mental health problems partially mediated the relationship between anxiety and employee performance. As a result, H11c was accepted and supported.

The value of R^2^ represents the proportion of variance in the dependent variable explained by its predictors (see Table 6). For mental health problems, job burnout, and employee performance, the three dependent variables in the research model, the R^2^ values were 0.619, 0.715, and 0.508, respectively. This shows that the five predictors (i.e., stress, depression, anxiety, job burnout, and mental health problems) accounted for 50.8% of the variance in employee performance. Overall, the R^2^ values were found to meet the cut-off value of 0.30 recommended by Sarfraz [11].

## 5. Discussion

The COVID-19 emergency has heavily affected the psychological condition of healthcare workers. Pakistan’s healthcare industry is at high risk of experiencing COVID-19 vulnerabilities [41]. COVID-19 has altered employees’ lives by causing them to experience unprecedented health consequences arising from their work. Healthcare employees are an organization’s most critical asset, able to enhance workplace productivity by delivering superior performance. To explain the effect of psychological problems on employee job performance, this discussion offers insights into the current study findings in light of previous literature. As such, the study seeks to provide an understanding of the effect of the COVID-19 emergency on front-line workers in Pakistan.

During the COVID-19 pandemic, organizations have reported negative consequences for healthcare performance. Kumar et al. [35] showed that COVID-19 induced stress in healthcare workers, inevitably undermining their job performance. Our study findings support previous studies that have demonstrated how COVID-19 stress significantly impacted healthcare performance [75]. These findings lead us to accept H1. Similarly, during the initial declaration of the COVID-19 pandemic, healthcare employees also reporting feeling depressive symptoms. Hennekam et al. [76] showed that increasing depression arising from the experience of COVID-19 caused healthcare workers to exhibit poor work performance. Previous research also indicated that COVID-19 intensified anxiety with negative effects on healthcare performance [77]. These studies support our findings, leading us to accept H2 and H3.

Several studies have illuminated the symptomatology of stress, anxiety, and depression during the pandemic. These psychological issues have deteriorated employees’ mental health and performance. Greenberg et al. [10] state that COVID-19-related psychological problems (e.g., stress, depression, and anxiety) have exerted unbearable pressure on workers’ mental health, influencing their work performance. A further consequence of the pandemic, and healthcare workers’ increased vulnerability to its effects, has been the growing incidence of burnout affecting healthcare performance. Poor health negatively affects employee performance. In this regard, Dyrbye et al. [78] found that many healthcare employees decided to quit their jobs due to increasing psychological problems during the pandemic. In the healthcare industry, these psychological problems (i.e., depression, stress, and anxiety) have drastically impeded workers’ performance, and increased the rate of job burnout [79]. Our study analysis, while supporting previous literature, has highlighted the mediating role of mental health and job burnout on employee performance. Overall, the study findings support the acceptance of all the proposed hypotheses and their assumptions.

This study was limited to healthcare employees working in three major cities of Pakistan, and future research could involve a comparative analysis of female and male healthcare workers’ job performance in public and private healthcare centers. The study employed cross-sectional data, but future research could consider longitudinal data. This study is based on a quantitative approach; in future research, mixed or qualitative approaches could be adopted. 

## 6. Conclusions

In recent years, the negative impact of COVID-19 has altered the professional lives of healthcare workers. The clinical features of COVID-19, including its infectivity, have placed a significant burden on employees’ mental health and performance. It has made front-line workers vulnerable, resulting in an increased level of psychological problems. 

The current study explored the extreme toll COVID-19 has taken on the healthcare industry of Pakistan. Significantly, the study results confirm the prevalence of stress, depression, and anxiety in front-line workers. The findings show that increasing COVID-19 stress and depression have caused employees to lose interest in their work tasks, thus decreasing their performance. Similarly, the results showed that COVID-19 anxiety significantly influenced employee performance. In addition, this review of the impact of the COVID-19 pandemic demonstrates that psychological issues (e.g., stress, depression, and anxiety) have influenced employees’ mental health and performance. Furthermore, the study has confirmed a significant mediating role of job burnout in influencing employee performance. 

Undoubtedly, the pandemic has made today’s employees vulnerable, and negatively impacted their workplace functioning. The literature shows that the impact of the pandemic drew the attention of researchers to individuals’ mental well-being. The results of the current study suggest that managers should respond to the adversity caused by the pandemic by ensuring superior job performance through improved mental health support. In conclusion, health professionals, practitioners, and policymakers should take steps to improve employees’ mental well-being and job performance.

## Figures and Tables

**Table 1:** Study participant’s demographic information.

Items	Frequency (N = 669)	(%)
Gender		
Male	311	46.5
Female	358	53.5
Age		
19–30	92	13.8
31–40	182	27.2
41–50	158	23.6
51–60	141	21.1
>60	96	14.3
Education		
Intermediate	130	19.4
Bachelor	216	32.3
Master	240	35.9
MPhil/Others	83	12.4
Occupation		
Nurses	310	46.3
Doctors	220	32.8
Technicians	90	13.4
Others	49	7.3
Marital Status		
Single	118	17.6
Married	551	82.4

**Table 2:** Model fit and reliability and validity analysis.

Model Fit Indexes					
Fit Index	Cited		Fit Criteria	Results	Fit (Yes/No)
X2				1454.113	
DF				1415	
X2/DF	Kline [61]		1.00–5.00	1.028	Yes
RMSEA	Steiger [62]		<0.08	0.006	Yes
SRMR	Hu & Bentler [63]		<0.08	0.0248	Yes
NFI	Bentler & G. Bonnet [64]		>0.80	0.935	Yes
IFI	Bollen [65]		>0.90	0.932	Yes
TLI	Tucker & Lewis [66]		>0.90	0.998	Yes
CFI	Byrne [67]		>0.90	0.998	Yes
GFI	Hoyle [68]		>0.90	0.929	Yes
Alpha, composite reliability and validity analysis					
Construct	Items	Loading	Alpha	CR	AVE
		>0.704	>0.7	>0.7	>0.5
Depression	DEP_1	0.721 ***	0.896	0.896	0.553
	DEP_2	0.739 ***			
	DEP_3	0.753 ***			
	DEP_4	0.745 ***			
	DEP_5	0.753 ***			
	DEP_6	0.749 ***			
	DEP_7	0.744 ***			
Stress	STR_1	0.715 ***	0.897	0.897	0.555
	STR_2	0.752 ***			
	STR_3	0.753 ***			
	STR_4	0.739 ***			
	STR_5	0.748 ***			
	STR_6	0.771 ***			
	STR_7	0.739 ***			
Anxiety	ANX_1	0.758 ***	0.902	0.902	0.567
	ANX_2	0.763 ***			
	ANX_3	0.753 ***			
	ANX_4	0.723 ***			
	ANX_5	0.763 ***			
	ANX_6	0.754 ***			
	ANX_7	0.758 ***			
Job Burnout	JBO_1	0.746 ***	0.773	0.773	0.532
	JBO_2	0.730 ***			
	JBO_3	0.713 ***			
Mental Health Problems	MHP_1	0.725 ***	0.945	0.945	0.533
	MHP_2	0.736 ***			
	MHP_3	0.753 ***			
	MHP_4	0.716 ***			
	MHP_5	0.742 ***			
	MHP_6	0.723 ***			
	MHP_7	0.757 ***			
	MHP_8	0.718 ***			
	MHP_9	0.729 ***			
	MHP_10	0.716 ***			
	MHP_11	0.754 ***			
	MHP_12	0.714 ***			
	MHP_13	0.736 ***			
	MHP_14	0.734 ***			
	MHP_15	0.697 ***			
Employee Performance	EP_1	0.721 ***	0.945	0.945	0.520
	EP_2	0.729 ***			
	EP_3	0.722 ***			
	EP_4	0.710 ***			
	EP_5	0.742 ***			
	EP_6	0.728 ***			
	EP_7	0.726 ***			
	EP_8	0.726 ***			
	EP_9	0.709 ***			
	EP_10	0.718 ***			
	EP_11	0.708 ***			
	EP_12	0.737 ***			
	EP_13	0.709 ***			
	EP_14	0.692 ***			
	EP_15	0.730 ***			
	EP_16	0.725 ***			

*** *p* < 0.001.

**Table 3:** Discriminant validity analysis (Fornell–Larcker and HTMT).

Constructs	Mean	SD	1	2	3	4	5	6
1. Depression	3.59	0.839	0.743	0.598	0.586	0.668	0.632	0.557
2. Stress	3.60	0.847	0.598	0.745	0.599	0.655	0.637	0.584
3. Anxiety	3.60	0.849	0.586	0.601	0.753	0.636	0.656	0.555
4. Job Burnout	3.62	0.886	0.666	0.653	0.636	0.730	0.681	0.587
5. Mental Health Problems	3.63	0.785	0.633	0.638	0.656	0.681	0.730	0.603
6. Employee Performance	3.65	0.608	−0.557	−0.584	−0.553	−0.587	−0.601	0.721

Note: Values on the diagonal (italicized) represent the square root of the average variance extracted, while the off diagonals are correlations.

**Table 4:** Hypotheses testing—direct effect.

Hypothesis	Direct	Std.	Std.	T	*p*
	Relationships	*Beta*	Error	Values	Values
H1	STR 🡺 EP	−0.205	0.037	−5.541	***
H2	DEP 🡺 EP	−0.123	0.041	−3.000	**
H3	ANX 🡺 EP	−0.113	0.039	−2.897	*
H4	STR 🡺 JBO	0.318	0.032	9.938	***
H5	DEP 🡺 JBO	0.368	0.031	11.871	***
H6	ANX 🡺 JBO	0.278	0.031	8.968	***
H7	STR 🡺 MHP	0.278	0.040	6.950	***
H8	DEP 🡺 MHP	0.279	0.037	7.541	***
H9	ANX 🡺 MHP	0.341	0.038	8.974	***
H10	JBO 🡺 EP	−0.195	0.050	−3.900	***
H11	MHP 🡺 EP	−0.183	0.053	−3.453	***

Indicates significant paths: * *p* < 0.05, ** *p* < 0.01, *** *p* < 0.001.

**Table 5:** Hypothesis results—indirect effects.

Hypothesis	Indirect	Std.	Lower	Upper	*p*
	Relationships	*Beta*	Limit	Limit	Values
H10a	STR 🡺 JBO 🡺 EP	−0.062	−0.058	−0.019	***
H10b	DEP 🡺 JBO 🡺 EP	−0.072	−0.077	−0.031	**
H10c	ANX 🡺 JBO 🡺 EP	−0.054	−0.059	−0.020	***
H11a	STR 🡺 MHP 🡺 EP	−0.051	−0.068	−0.027	**
H11b	DEP 🡺 MHP 🡺 EP	−0.051	−0.058	−0.022	**
H11c	ANX 🡺 MHP 🡺 EP	−0.063	−0.071	−0.021	**

Indicates significant paths: ** *p* < 0.01, *** *p* < 0.001.

**Table 6:** R^2^ Values.

Latent Variables	R^2^
MHP	0.619
JBO	0.715
EP	0.508

## Data Availability

The current study data can be obtained from the corresponding author.

## References

[1] Silva P.C., Batista P.V., Lima H.S., Alves M.A., Guimarães F.G., Silva R.C. (2020). COVID-ABS: An agent-based model of COVID-19 epidemic to simulate health and economic effects of social distancing interventions. Chaos Solitons Fractals.

[2] Xiao C. (2020). A novel approach of consultation on 2019 novel coronavirus (COVID-19)-related psychological and mental problems: Structured letter therapy. Psychiatry Investig..

[3] Nicola M., Alsafi Z., Sohrabi C., Kerwan A., Al-Jabir A., Iosifidis C., Agha M., Agha R. (2020). The socio-economic implications of the coronavirus pandemic (COVID-19): A review. Int. J. Surg..

[4] Razu S.R., Yasmin T., Arif T.B., Islam S., Islam S.M.S., Gesesew H.A., Ward P. (2021). Challenges Faced by Healthcare Professionals during the COVID-19 Pandemic: A Qualitative Inquiry from Bangladesh. Front. Public Health.

[5] Khan A.A., Lodhi F.S., Rabbani U., Ahmed Z., Abrar S., Arshad S., Khan M.I. (2021). Impact of coronavirus disease (COVID-19) pandemic on psychological well-being of the Pakistani general population. Front. Psychiatry.

[6] Zheng W. (2020). Mental health and a novel coronavirus (2019-nCoV) in China. J. Affect. Disord..

[7] Xiang Y.-T., Yang Y., Li W., Zhang L., Zhang Q., Cheung T., Ng C.H. (2020). Timely mental health care for the 2019 novel coronavirus outbreak is urgently needed. Lancet Psychiatry.

[8] Sun J., Sarfraz M., Khawaja K.F., Ozturk I., Raza M.A. (2022). The Perils of the Pandemic for the Tourism and Hospitality Industries: Envisaging the Combined Effect of COVID-19 Fear and Job Insecurity on Employees’ Job Performance in Pakistan. Psychol. Res. Behav. Manag..

[9] Muller A.E., Hafstad E.V., Himmels J.P.W., Smedslund G., Flottorp S., Stensland S.Ø., Stroobants S., Van de Velde S., Vist G.E. (2020). The mental health impact of the COVID-19 pandemic on healthcare workers, and interventions to help them: A rapid systematic review. Psychiatry Res..

[10] Greenberg N., Docherty M., Gnanapragasam S., Wessely S. (2020). Managing mental health challenges faced by healthcare workers during COVID-19 pandemic. BMJ.

[11] Sarfraz M., Ji X., Asghar M., Ivascu L., Ozturk I. (2022). Signifying the Relationship between Fear of COVID-19, Psychological Concerns, Financial Concerns and Healthcare Employees Job Performance: A Mediated Model. Int. J. Environ. Res. Public Health.

[12] Fernandez R., Sikhosana N., Green H., Halcomb E.J., Middleton R., Alananzeh I., Trakis S., Moxham L. (2021). Anxiety and depression among healthcare workers during the COVID-19 pandemic: A systematic umbrella review of the global evidence. BMJ Open.

[13] Aguiar-Quintana T., Nguyen T.H.H., Araujo-Cabrera Y., Sanabria-Díaz J.M. (2021). Do job insecurity, anxiety and depression caused by the COVID-19 pandemic influence hotel employees’ self-rated task performance? The moderating role of employee resilience. Int. J. Hosp. Manag..

[14] Zahra M., Akhtar A., Arzoo H., Humayun S., Aman H., Andleeb S.N. (2021). Exploring Stress Coping Strategies of Front-Line Emergency Medical Experts Dealing COVID-19 in Pakistan: A Qualitative Inquiry. Multicult. Educ..

[15] Wang L., Wang H., Shao S., Jia G., Xiang J. (2020). Job Burnout on Subjective Well-Being among Chinese Female Doctors: The Moderating Role of Perceived Social Support. Front. Psychol..

[16] Guixia L., Hui Z. (2020). A Study on Burnout of Nurses in the Period of COVID-19. Psychol. Behav. Sci..

[17] Yunita P.I., Saputra I.G.N.W.H. (2019). Millennial generation in accepting mutations: Impact on work stress and employee performance. Int. J. Soc. Sci. Humanit..

[18] Prasada K.D.V., Vaidyab R.W., Mangipudic M.R. (2020). Effect of occupational stress and remote working on psychological well-being of employees: An empirical analysis during COVID-19 pandemic concerning information technology industry in hyderabad. Indian J. Commer. Manag. Stud..

[19] Darvishmotevali M., Ali F. (2020). Job insecurity, subjective well-being and job performance: The moderating role of psychological capital. Int. J. Hosp. Manag..

[20] Tu Y., Li D., Wang H.-J. (2021). COVID-19-induced layoff, survivors’ COVID-19-related stress and performance in hospitality industry: The moderating role of social support. Int. J. Hosp. Manag..

[21] Galbraith N., Boyda D., McFeeters D., Hassan T. (2021). The mental health of doctors during the COVID-19 pandemic. BJPsych Bull..

[22] Deng J., Guo Y., Ma T., Yang T., Tian X. (2019). How job stress influences job performance among Chinese healthcare workers: A cross-sectional study. Environ. Health Prev. Med..

[23] Wang C., Pan R., Wan X., Tan Y., Xu L., Ho C.S., Ho R.C. (2020). Immediate Psychological Responses and Associated Factors during the Initial Stage of the 2019 Coronavirus Disease (COVID-19) Epidemic among the General Population in China. Int. J. Environ. Res. Public Health.

[24] Gu Y., Guo J., Liao L., Wang B., Li X., Guo L., Tong Z., Guan Q., Zhou M., Wu Y. (2021). Psychological effects of COVID-19 on hospital staff: A national cross-sectional survey in mainland China. Vasc. Investig. Ther..

[25] Heath C., Sommerfield A., Von Ungern-Sternberg B.S. (2020). Resilience strategies to manage psychological distress among healthcare workers during the COVID-19 pandemic: A narrative review. Anaesthesia.

[26] Hadi S.A., Bakker A.B., Häusser J.A. (2021). The role of leisure crafting for emotional exhaustion in telework during the COVID-19 pandemic. Anxiety Stress Coping.

[27] Sarfraz M., Hafeez H., Abdullah M.I., Ivascu L., Ozturk I. (2022). The effects of the COVID-19 pandemic on healthcare workers’ psychological and mental health: The moderating role of felt obligation. Work.

[28] Parent-Lamarche A., Marchand A., Saade S. (2020). Does Depression Mediate the Effect of Work Organization Conditions on Job Performance?. J. Occup. Environ. Med..

[29] Khawaja K.F., Sarfraz M., Rashid M., Rashid M. (2022). How is COVID-19 pandemic causing employee withdrawal behavior in the hospitality industry? An empirical investigation. J. Hosp. Tour. Insights.

[30] Gao J., Zheng P., Jia Y., Chen H., Mao Y., Chen S., Wang Y., Fu H., Dai J. (2020). Mental health problems and social media exposure during COVID-19 outbreak. PLoS ONE.

[31] Ullah I., Khan K.S., Ali I., Ullah A.R., Mukhtar S., de Filippis R., Malik N.I., Shalbafan M., Hassan Z., Asghar M.S. (2022). Depression and anxiety among Pakistani healthcare workers amid COVID-19 pandemic: A qualitative study. Ann. Med. Surg..

[32] Khalid A., Ali S. (2020). COVID-19 and its Challenges for the Healthcare System in Pakistan. Asian Bioeth. Rev..

[33] Fu S.Q., Greco L.M., Lennard A.C., Dimotakis N. (2021). Anxiety responses to the unfolding COVID-19 crisis: Patterns of change in the experience of prolonged exposure to stressors. J. Appl. Psychol..

[34] De Clercq D., Azeem M.U., Haq I.U. (2020). But they promised! How psychological contracts influence the impact of felt violations on job-related anxiety and performance. Pers. Rev..

[35] Kumar P., Kumar N., Aggarwal P., Yeap J.A.L. (2021). Working in lockdown: The relationship between COVID-19 induced work stressors, job performance, distress, and life satisfaction. Curr. Psychol..

[36] Nadeem F., Sadiq A., Raziq A., Iqbal Q., Haider S., Saleem F., Bashaar M. (2021). Depression, Anxiety, and Stress among Nurses During the COVID-19 Wave III: Results of a Cross-Sectional Assessment. J. Multidiscip. Health.

[37] Mamo E., Tadesse T., Kibret A., Weldeyohanes G., Yesuf A., Endazenaw G. (2022). Burn out among Healthcare Workers (HCWs) at Middle Stage of COVID-19 Pandemic in Addis Ababa, Ethiopia: A Multicentre Cross-Sectional Study. Pathol. Lab. Med..

[38] Pfefferbaum B., North C.S. (2020). Mental Health and the COVID-19 Pandemic. N. Engl. J. Med..

[39] Bradley M., Chahar P. (2020). Burnout of healthcare providers during COVID-19. Cleve. Clin. J. Med..

[40] Ahmad S., Yaqoob S., Safdar S., Cheema H.A., Islam Z., Iqbal N., Tharwani Z.H., Swed S., Ijaz M.S., Rehman M.U. (2022). Burnout in health care workers during the fourth wave of COVID-19: A cross sectional study from Pakistan. Ann. Med. Surg..

[41] Zahid N., Syed M., Hersi S., Danish S.H., Ahmed F. (2021). Assessment of Burnout in Healthcare Professionals of Pakistan Amid COVID-19—A Cross-Sectional Study. Int. J. Trop. Dis. Health.

[42] Javed B., Sarwer A., Soto E.B., Mashwani Z.-R. (2020). Is Pakistan’s Response to Coronavirus (SARS-CoV-2) Adequate to Prevent an Outbreak?. Front. Med..

[43] Çelmeçe N., Menekay M. (2020). The Effect of Stress, Anxiety and Burnout Levels of Healthcare Professionals Caring for COVID-19 Patients on Their Quality of Life. Front. Psychol..

[44] Abdullah M.I., Huang D., Sarfraz M., Ivascu L., Riaz A. (2021). Effects of internal service quality on nurses’ job satisfaction, commitment and performance: Mediating role of employee well-being. Nurs. Open.

[45] Kok N., van Gurp J., Teerenstra S., van der Hoeven H., Fuchs M., Hoedemaekers C., Zegers M. (2021). Coronavirus Disease 2019 Immediately Increases Burnout Symptoms in ICU Professionals: A Longitudinal Cohort Study. Crit. Care Med..

[46] Sharifi M., Asadi-Pooya A.A., Mousavi-Roknabadi1 R.S. (2021). Burnout among Healthcare Providers of COVID-19; a Systematic Review of Epidemiology and Recommendations. Arch. Acad. Emerg. Med..

[47] Saleem F., Malik M.I., Qureshi S.S. (2021). Work Stress Hampering Employee Performance during COVID-19: Is Safety Culture Needed?. Front. Psychol..

[48] Hofmeyer A., Taylor R., Kennedy K. (2020). Fostering compassion and reducing burnout: How can health system leaders respond in the COVID-19 pandemic and beyond?. Nurse Educ. Today.

[49] Teo I., Chay J., Cheung Y.B., Sung S.C., Tewani K.G., Yeo L.F., Yang G.M., Pan F.T., Ng J.Y., Aloweni F.A.B. (2021). Healthcare worker stress, anxiety and burnout during the COVID-19 pandemic in Singapore: A 6-month multi-centre prospective study. PLoS ONE.

[50] Naldi A., Vallelonga F., Di Liberto A., Cavallo R., Agnesone M., Gonella M., Sauta M.D., Lochner P., Tondo G., Bragazzi N.L. (2021). COVID-19 pandemic-related anxiety, distress and burnout: Prevalence and associated factors in healthcare workers of North-West Italy. BJPsych Open.

[51] Pappa S., Ntella V., Giannakas T., Giannakoulis V.G., Papoutsi E., Katsaounou P. (2020). Prevalence of depression, anxiety, and insomnia among healthcare workers during the COVID-19 pandemic: A systematic review and meta-analysis. Brain Behav. Immun..

[52] Satici B., Gocet-Tekin E., Deniz M.E., Satici S.A. (2021). Adaptation of the Fear of COVID-19 Scale: Its Association with Psychological Distress and Life Satisfaction in Turkey. Int. J. Ment. Health Addict..

[53] Brooks S.K., Webster R.K., Smith L.E., Woodland L., Wessely S., Greenberg N., Rubin G.J. (2020). The psychological impact of quarantine and how to reduce it: Rapid review of the evidence. Lancet.

[54] Lei L., Huang X., Zhang S., Yang J., Yang L., Xu M. (2020). Comparison of Prevalence and Associated Factors of Anxiety and Depression among People Affected by versus People Unaffected by Quarantine during the COVID-19 Epidemic in Southwestern China. Med. Sci. Monit..

[55] Lai J., Ma S., Wang Y., Cai Z., Hu J., Wei N., Wu J., Du H., Chen T., Li R. (2020). Factors associated with mental health outcomes among health care workers exposed to coronavirus disease 2019. JAMA Netw. Open.

[56] Podsakoff P.M., MacKenzie S.B., Lee J.-Y., Podsakoff N.P. (2003). Common method biases in behavioral research: A critical review of the literature and recommended remedies. J. Appl. Psychol..

[57] Vignola R.C.B., Tucci A.M. (2014). Adaptation and validation of the depression, anxiety and stress scale (DASS) to Brazilian Portuguese. J. Affect. Disord..

[58] Ninaus K., Diehl S., Terlutter R. (2021). Employee perceptions of information and communication technologies in work life, perceived burnout, job satisfaction and the role of work-family balance. J. Bus. Res..

[59] Sharma P., Devkota G. (2019). Mental health screening questionnaire: A study on reliability and correlation with perceived stress score. J. Psychiatr. Assoc. Nepal.

[60] Ferozi S., Chang Y. (2021). Transformational Leadership and its Impact on Employee Performance: Focus on Public Employees in Afghanistan. Transylv. Rev. Adm. Sci..

[61] Kline R.B. (2010). Principles and Practice for Structural Equation Modelling.

[62] Steiger J.H. (1990). Structural model evaluation and modification: An interval estimation approach. Multivar. Behav. Res..

[63] Hu L., Bentler P.M. (1999). Cutoff criteria for fit indexes in covariance structure analysis: Conventional criteria versus new alternatives. Struct. Equ. Modeling A Multidiscip. J..

[64] Bentler P.M., Bonett D.G. (1980). Significance tests and goodness of fit in the analysis of covariance structures. Psychol. Bull..

[65] Bollen K.A. (1990). Overall fit in covariance structure models: Two types of sample size effects. Psychol. Bull..

[66] Tucker L.R., Lewis C. (1973). A reliability coefficient for maximum likelihood factor analysis. Psychometrika.

[67] Byrne B.M. (2016). Structural Equation Modeling with AMOS: Basic Concepts, Applications, and Programming.

[68] Hoyle R.H. (1991). Evaluating measurement models in clinical research: Covariance structure analysis of latent variable models of self-conception. J. Consult. Clin. Psychol..

[69] Hair J., Anderson R., Mehta R., Babin B. (2008). Sales Management: Building Customer Relationships and Partnerships.

[70] Nunnally J.C. (1994). Psychometric Theory 3E.

[71] Bagozzi R.P., Yi Y. (1988). On the evaluation of structural equation models. J. Acad. Mark. Sci..

[72] Kline T. (2005). Psychological Testing: A Practical Approach to Design and Evaluation.

[73] Fornell C., Larcker D.F. (1981). Evaluating structural equation models with unobservable variables and measurement error. J. Mark. Res..

[74] Hair F.H., Hult G.T.M., Ringle C., Sarstedt M. (2016). A Primer on Partial Least Squares Structural Equation Modeling (PLS-SEM).

[75] Wong A.K.F., Kim S.S., Kim J., Han H. (2021). How the COVID-19 pandemic affected hotel Employee stress: Employee perceptions of occupational stressors and their consequences. Int. J. Hosp. Manag..

[76] Hennekam S., Richard S., Grima F. (2020). Coping with mental health conditions at work and its impact on self-perceived job performance. Empl. Relations Int. J..

[77] Labrague L.J., Santos J.A.A. (2020). COVID-19 anxiety among front-line nurses: Predictive role of organisational support, personal resilience and social support. J. Nurs. Manag..

[78] Dyrbye L.N., Shanafelt T.D., Johnson P.O., Johnson L.A., Satele D., West C.P. (2019). A cross-sectional study exploring the relationship between burnout, absenteeism, and job performance among American nurses. BMC Nurs..

[79] Sun L., Wang G., Gao L. (2022). Modelling the Impact of Tourism on Mental Health of Chinese Residents: An Empirical Study. Discret. Dyn. Nat. Soc..

